# Effectiveness of a web-based treatment program using intensive therapeutic support for female patients with bulimia nervosa, binge eating disorder and eating disorders not otherwise specified: study protocol of a randomized controlled trial

**DOI:** 10.1186/1471-244X-13-310

**Published:** 2013-11-16

**Authors:** Elke D ter Huurne, Marloes G Postel, Hein A de Haan, Cor AJ DeJong

**Affiliations:** 1 Tactus Addiction Treatment, Institutenweg1, P.O. Box 154, Enschede, PH, 7521, The Netherlands; 2 Nijmegen Institute for Scientist Practitioners in Addiction, Toernooiveld 5, P.O. Box 6909, 6503 GK Nijmegen, The Netherlands; 3 Department of Psychology Health & Technology, University of Twente, P.O. Box 217, 7500 AE Enschede, The Netherlands; 4 Behavioural Science Institute, Radboud University Nijmegen, Montessorilaan 3, P.O. Box 9104, 6500 HE Nijmegen, The Netherlands

**Keywords:** Eating disorders, Bulimia nervosa, Binge eating disorder, Eating disorders not otherwise specified, Randomized controlled trial, Web-based treatment program, e-health, Intensive therapeutic support, Asynchronous support

## Abstract

**Background:**

Disordered eating behavior and body dissatisfaction affect a large proportion of the Dutch population and account for severe psychological, physical and social morbidity. Yet, the threshold for seeking professional care is still high. In the Netherlands, only 7.5% of patients with bulimia nervosa and 33% of patients with anorexia nervosa are treated within the mental health care system. Easily accessible and low-threshold interventions, therefore, are needed urgently. The internet has great potential to offer such interventions. The aim of this study is to determine whether a web-based treatment program for patients with eating disorders can improve eating disorder psychopathology among female patients with bulimia nervosa, binge eating disorder and eating disorders not otherwise specified.

**Methods/design:**

This randomized controlled trial will compare the outcomes of an experimental treatment group to a waiting list control group. In the web-based treatment program, participants will communicate personally and asynchronously with their therapists exclusively via the internet. The first part of the program will focus on analyzing eating attitudes and behaviors. In the second part of the program participants will learn how to change their attitudes and behaviors. Participants assigned to the waiting list control group will receive no-reply email messages once every two weeks during the waiting period of 15 weeks, after which they can start the program. The primary outcome measure is an improvement in eating disorder psychopathology as determined by the Eating Disorder Examination Questionnaire. Secondary outcomes include improvements in body image, physical and mental health, body weight, self-esteem, quality of life, and social contacts. In addition, the participants’ motivation for treatment and their acceptability of the program and the therapeutic alliance will be measured. The study will follow the recommendations in the CONSORT statement relating to designing and reporting on RCTs.

**Discussion:**

This study protocol presents the design of a RCT for evaluating the effectiveness of a web-based treatment program using intensive therapeutic support for female patients with bulimia nervosa, binge eating disorder and eating disorders not otherwise specified.

**Trial registration:**

The protocol for this study is registered with the Netherlands Trial Registry NTR2415.

## Background

A significant part of the Dutch population suffers from disordered eating behavior and body dissatisfaction. According to a recent review on the epidemiology of eating disorders, the incidence of anorexia nervosa (AN) in the Netherlands over the past decade has remained stable at around 7 per 100,000 persons per year [1]. For bulimia nervosa (BN) the incidence rate has been estimated at 6.1 per 100,000 persons per year [1]. The latest incidence numbers of AN and BN in the Netherlands are, however, from a national primary care study dated 1995–1999 [1]. In a population-based study in six European countries, including the Netherlands, the lifetime prevalence of AN and BN was estimated to be 0.48% and 0.51%, respectively [2]. For the Netherlands, the lifetime prevalence estimates were somewhat lower compared to the other countries (except for Germany) [2]. In addition to AN and BN, the most common eating disorder diagnosis is the category 'eating disorders not otherwise specified’ (EDNOS), including binge eating disorder (BED) [3,4]. In the six European countries studied, the lifetime prevalence of BED was 1.12% [2].

Eating disorders can have severe psychological, physical and social consequences, and clearly affect the quality of life of the patient [5-7]. Despite all the negative consequences, many patients do not seek or receive mental health care [8-10]. Barriers that prevent patients from seeking help include feelings of shame, fear of stigma, a lack of awareness, denial of the disorder, low motivation for treatment, availability of health services, mistrust in the system, and the high cost [11-15]. According to a review by Smink, van Hoeken & Hoek, only 7.5% of all patients with BN and 33% of all patients with AN are treated within the mental health care system [1]. The threshold for seeking professional care is high. Consequently, there is an urgent need for easily accessible and low-threshold interventions.

The internet with its widespread access and increasing usage, offers a great opportunity to administer appealing and accessible interventions for patients with eating disorders. Internet interventions can offer a high degree of anonymity that may encourage patients who are ashamed of their eating problems to seek help. They can also offer instant and easy access to treatment for patients living in rural and remote locations [16-19]. Newly developed internet interventions have already been applied successfully for various mental health illnesses, including alcohol abuse [20-22], depression [23-25] and anxiety disorders [24,26-28].

Several studies have been conducted into web-based interventions designed to prevent eating disorders [29,30]. However, the number of studies that focus on the treatment of eating disorders through web-based interventions is rather limited. A recently conducted systematic review on internet-based treatment of eating disorders [31] found that such treatments were more effective than waiting lists in reducing eating disorder psychopathology, frequency of binge eating and purging, and in improving quality of life. However, according to the authors, the methodological quality of the studies varied. The internet-based treatments the review investigated differed considerably in the intensity of therapeutic support [31]. Some studies involved self-help or minimal contact interventions, whereas others included weekly contact between participants and their coaches or licensed therapists. Research in the field of depression and anxiety has shown that interventions using (intensive) therapeutic support are more effective than self-help or minimal contact interventions [24,32-34]. For web-based treatments on eating disorders, however, similar research is scarce. According to Aardoom and colleagues, face-to-face contacts and therapeutic support seem to increase study compliance and possibly also treatment compliance, as therapist support appears to play an important role in enhancing participants’ motivation [31]. However, more research is needed on the role of therapeutic support in web-based treatments on eating disorders, with regard to the optimal frequency, amount, type and provider of support.

In the Netherlands, several mental health care institutions provide web-based treatments for patients with eating disorders. As far as we know, only two of these interventions have been studied to date [35-37], and just one of them was conducted among the Dutch population of patients with eating disorders (specifically BN) [37]. This study included a randomized controlled trial (RCT) on an asynchronous web-based treatment program with intensive therapeutic support (25 scheduled clinician feedback moments) among Dutch patients with BN. The RCT showed that the intervention was effective in decreasing disordered eating behavior (measured with the EDE-Q; *d* = 1.5, p <0.001) and body dissatisfaction (measured with the BAT; *d* = 0.7, p <0.001) [37]. Also, the frequency of binge eating and purging reduced from 15 and 14, respectively, at pre-test, to 2 and 0, respectively, at post-test (both p <0.001).

So far, the effects of web-based treatments have not been analyzed for patients with BED and EDNOS in the Netherlands. Given the high prevalence of these eating disorders, it would be interesting to investigate whether a web-based intervention would also be appropriate and effective for these patient groups. Therefore, a web-based intervention with intensive therapeutic support was developed for Dutch patients with all types of eating disorders, including AN, BN, EDNOS and BED [38]. The design and content of the intervention were based on a comparable, successful web-based intervention for problem drinkers [20,39].

In 2010, a pilot study tested the web-based intervention with 165 patients with eating disorders to determine its effectiveness and feasibility [40]. A clinically relevant improvement between baseline and post treatment was found for eating disorder psychopathology (measured with EDE-Q, *d* = 1.17, p < .001) and body dissatisfaction (measured with BAT; *d* = 0.86, p <0.001). Also quality of life (measured with the EuroQol-5D [EQ-5D], *d* = 0.32, p < .001), mental health (measured with the Depression Anxiety Stress Scale [DASS], *d* = 0.56, p < .001), and physical health (measured with the Maudsley Addiction Profile Health Symptom Scale [MAP-HSS] and 15 additional eating disorder-specific complaints, *d* = 0.39, p < .001) clearly improved. All of the effects, with the exception of improvement in quality of life, were sustained at six-weeks and six-months follow-up, and participants’ satisfaction with the treatment program was high. However, the pilot study had a nonrandomized design, so the observed improvements could not be attributed exclusively to the web-based treatment program. Furthermore, 46% of the participants ended the web-based treatment program prematurely and no post treatment and follow-up data were available from these participants. Therefore, it was unknown whether they benefited from participating in the intervention. Accordingly, further research into the efficacy of the web-based treatment program and into the group of those who do not complete the program is needed.

### Study aims

The primary aim of this study is to evaluate whether a Dutch web-based treatment program with intensive therapeutic support can improve eating disorder psychopathology among female patients with BN, BED and EDNOS. The secondary aim is to explore whether the treatment program can also improve body image, physical health, body weight, mental health, self-esteem, quality of life, and social contacts. Additional aims include examining participants’ acceptability of the web-based treatment program, adherence to the program, reasons for non-completion, the long-term treatment effects 3, 6 and 12 months after completing treatment, the influence of motivation on treatment adherence and treatment effectiveness, the therapeutic alliance between the participants (completers as well as non-completers) and their therapists, the predictors of treatment effectiveness and treatment adherence, and the additional value of participating in the online forum on the effect of treatment and adherence to it. In line with the conclusions of the review by Aardoom and colleagues [31], the involvement of other interventions during treatment and follow-up, as well as changes in medication use during treatment, will also be monitored and reported.

## Methods/design

### Study design

**Figure 1:**
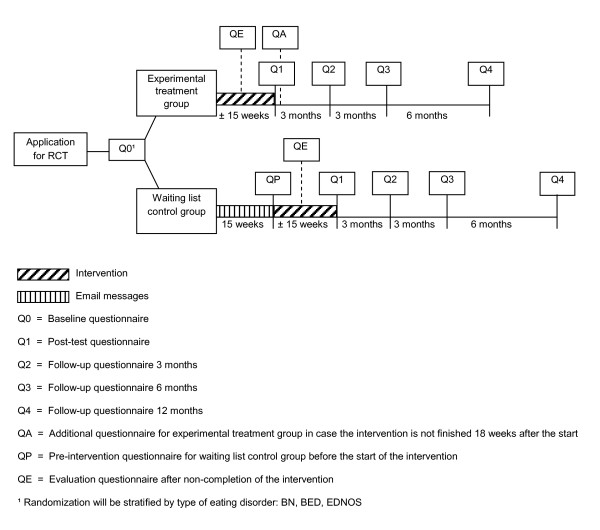
Flow diagram of the study.

The present study is a parallel-arm randomized controlled trial that will compare the outcomes of participants assigned to the experimental treatment group (web-based treatment program) with those assigned to the waiting list control group. Figure 1 presents a flow diagram of the study.

Baseline data for both groups will be obtained from the baseline questionnaire (Q0). After randomization, the participants in the experimental treatment group will immediately start with the web-based treatment program while the participants assigned to the control group will receive informational and supportive no-reply email messages once every two weeks until they can start with the web-based treatment program 15 weeks later. A waiting period of 15 weeks was chosen, as this was the average period of treatment in our pilot study and we assume this will also be the case in the present study. Before the participants in the control group can begin the web-based treatment program, they will be asked to complete the pre-intervention questionnaire (QP) to determine whether there are any differences between the baseline situation and the situation after the 15-week waiting period.

Each participant in the experimental treatment group will receive the post-test questionnaire (Q1) after completing the web-based treatment program. The post-test questionnaire will measure primary and secondary outcomes and the participant’s acceptability of the web-based treatment program. In cases where participants in the experimental treatment group do not fully complete the web-based treatment program within 18 weeks, they will receive the additional questionnaire (QA) 18 weeks after the start of the intervention. This questionnaire will replace the post-test questionnaire (Q1) as the second assessment, to set the period between the first and second assessments as close as possible in both groups. Assessment QA is identical to the post-test questionnaire (Q1), with the exception of the questions to evaluate the web-based treatment program.

The participants in both groups will receive a follow-up questionnaire three, six, and 12 months after completing the treatment program (assessments Q2, Q3 and Q4, respectively).

Each participant who ends the web-based treatment program prematurely will receive the evaluation questionnaire (QE) instead of the post-test questionnaire (Q1). The evaluation questionnaire measures the participant’s reasons for non-completing and the acceptability of the web-based treatment program. In addition, the primary and secondary outcomes will be measured. Each participant will also receive the follow-up questionnaires (Q2, Q3 and Q4).

As a reward for completing the extensive questionnaires, each participant will receive a €10.00 digital coupon to an online store (http://www.bol.com) for each completed research questionnaire. The baseline questionnaire (Q0) is an exception, as this questionnaire is primarily relevant for the therapist, whereas the other questionnaires are primarily relevant for research purposes. Consequently, each participant can receive a maximum of €50.00 for completing all of the research questionnaires.

### Setting

The participants’ personal setting can be any environment of their choice that has internet access. In 2011, 94% of the Dutch population had internet access in their homes, and approximately 87% used the internet (almost) every day [41]. In this way, patients throughout the country can participate in the study. The therapists’ setting will be the department 'Web-based interventions’ of Tactus Addiction Treatment, which is located in Enschede, the Netherlands.

### Participants

The study population will consist of female patients with BN, BED and EDNOS. Based on the results of our pilot study, we will exclude males and patients with AN, as these patient groups were a minority in our pilot study. Male patients and patients with AN will be offered the possibility of participating in the regular web-based treatment program without participating in the RCT.

### Inclusion criteria

The inclusion criteria for the present study are: 1) female; 2) aged 18 years or older; 3) a diagnosis of BN, BED or EDNOS; 4) access to and ability to use the internet; 5) the ability to read and write Dutch; and 6) have given informed consent.

### Exclusion criteria

The exclusion criteria for the study are: 1) body weight less than 85% of ideal body weight; 2) other psychological and/or pharmaceutical treatment for eating disorders in the past six months; 3) critical suicidal ideation (suicidal plans or attempted suicide in the past month, or attempted suicide once and suicidal thoughts in the past month); 4) being pregnant; and 5) planning to be absent for at least four weeks during the intervention period.

### Recruitment

For recruitment, self-selection through a placed advertisement will be used. Information regarding the web-based treatment program and the trial can be found on the Dutch website http://www.etendebaas.nl and on other Dutch eating disorder-related websites and forums. We will also place targeted advertisements in Dutch newspapers and attempt to obtain free publicity for the intervention and the study in newspapers and magazines.

### Procedure

Participants will receive an email with an attachment containing a description (in Dutch) of the research project and the web-based intervention. Written informed consent must be provided by each participant prior to randomization. The consent form will include information regarding withdrawal of consent, providing personal information, and GP data.

Each participant will also complete the baseline questionnaire (Q0), which is an extensive online questionnaire consisting of questions regarding eating attitudes and behaviors, body dissatisfaction, physical and mental health, and the personal situation of the participant. Based on the replies given in the baseline questionnaire, the investigator will evaluate whether the participant meets the inclusion or exclusion criteria of the study. An eating disorder diagnosis will be determined using an online self-report questionnaire based on the DSM-IV criteria for BN and the criteria described in Appendix B of the DSM-IV-TR for BED. The Dutch version of the Mini-International Neuropsychiatric Interview–Plus (MINI-Plus) [42,43] will be used as a guideline. The MINI-Plus is an appropriate measurement for daily psychiatric practice and research purposes to determine DSM-IV diagnoses in a standardized way [44]. Participants with one or more eating disorder characteristics who do not meet the criteria of BN or BED will be diagnosed with EDNOS. An exception will be made for those participants who meet the exclusion criterion “body weight less than 85% of ideal weight” which will be measured using the weight table in the MINI-Plus part N (Anorexia Nervosa) [43,44]. To assess this exclusion criterion, the participants will be asked for their height and lowest body weight in the three months prior to the study.

Participants who do not meet the inclusion criteria and are therefore not eligible to participate in this study will be informed of the possibility of participating in the standard web-based treatment program without having to participate in this study. Participants who cannot participate in the web-based treatment program (for example, because they already receive treatment for their eating disorder at another institution or due to urgent medical reasons) will be referred to their GP or to a mental health institution.

### Study intervention

#### Experimental treatment group

The web-based treatment program is part of an online application for patients with eating disorders. The application also includes an informational website (http://www.etendebaas.nl) and a forum for peer support [38,40]. The contents and elements of the treatment program are based on two evidence-based techniques: Cognitive Behavioral Therapy [45-47] and Motivational Interviewing [48,49].

During the web-based treatment program, all participants will have their own therapist with whom they will communicate personally and asynchronously twice a week, solely through the internet. There will be no face-to-face or telephone contact during the treatment period, unless participants specifically request this. The asynchronous communication resembles email contact, but will take place within a secure web-based application. Participants will receive a response from their therapist within three working days. Asynchronous contact was chosen because it will give participants more autonomy to decide when to participate in the treatment program, and it enables them to think carefully about the responses they will give to the therapist. The intensive and personalized interaction between participants and their therapists is one of the most essential elements of the web-based treatment program and makes this intervention stand out from online self-help programs and minimal-contact therapies. The aim of the intense interaction is to optimize the relationship between the participants and their therapists, as the therapeutic alliance is positively associated with treatment outcome in face-to-face treatment [50,51] as well as web-based treatment [52,53]. However, so far the therapeutic relationship has not been studied extensively for web-based treatments on eating disorders [31].

**Figure 2:**
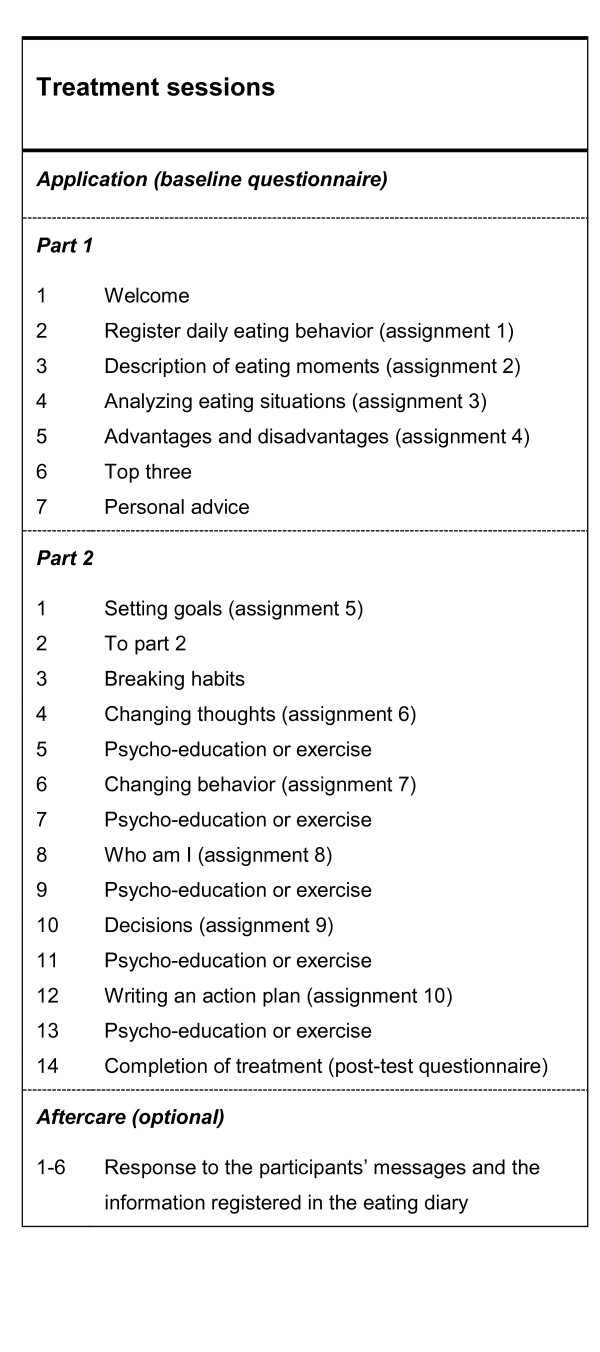
Treatment sessions of the web-based treatment program.

All of the therapists working on the web-based treatment program will have a bachelor’s degree in nursing or social work or a master’s degree in psychology. Furthermore, they will complete an intensive two-day training program that focuses on motivational writing skills, treatment methods for patients with eating disorders, implementation of the treatment protocol, and the technical aspects of delivering this web-based intervention. Subsequently, all therapists will complete a full treatment program with a test patient, after which they can start under supervision as online therapists. Intensive supervision by highly experienced coaches will continue for at least three months, during which a coach will provide feedback on all therapists’ messages before they are sent to the participants. These participants may join the treatment program within their own personal environment at any time that suits them. Therefore, no pre-determined appointments will be required. The average duration of the web-based treatment program will be 15 weeks, with two contacts a week. Part 1 of the program will consist of four assignments and at least seven contacts between the participant and the therapist, with a focus on analyzing the eating attitudes and behaviors of the participant. At the end of Part 1, the participant will receive personal advice from the therapist. Part 2 involves six assignments and at least 14 contacts. This part of the treatment program will start with setting goals for eating behavior, exercising, weighing, and compensatory behaviors (if applicable). The participants will learn to reach these goals in five steps. In addition to receiving assignments, the participants will also receive psycho education and exercises. After completing the program, participants may take part in an aftercare program consisting of six weekly sessions. The purpose of this program will be to support participants in their efforts to maintain the changes achieved through the treatment program. During the sessions, the therapist will respond to participants’ messages and the information registered in the eating diary. Besides the aftercare sessions, there will be three follow-up contacts, 3, 6 and 12 months after completion of the treatment, in which the therapist will ask the participant to complete the follow-up questionnaire. The treatment sessions are presented in Figure 2.

All Dutch citizens have mandatory health care coverage. Dutch health insurance companies will reimburse costs participants incur by taking part in this web-based treatment program, with the possible exemption of a participant contribution up to 350 euro.

#### Waiting list control group (no-reply e-mail messages)

The participants in the waiting list control group will have to wait for 15 weeks after the randomization before they can start taking part in the web-based intervention. During this period, the participants will receive a no-reply email from the researcher every two weeks to keep them involved in the study. To avoid therapeutic support, the participants in the waiting list control group cannot reply to these messages. The email messages will include information about eating behaviors and body image, psycho education, motivational messages, and references to the informational website and online forum.

### Primary outcome measure

Primary outcome measure for this study will be an improvement in the eating disorder psychopathology. This will be measured with the Eating Disorder Examination Questionnaire (EDE-Q) [54,55]. The EDE-Q is a 36-item self-report questionnaire, derived from the Eating Disorder Examination Interview (EDE) [56]. The EDE-Q is widely used to assess the key behavioral features of eating disorders and the severity of the psychopathology of eating disorders. The EDE-Q consists of four subscales: Restraint, Eating Concern, Shape Concern, and Weight Concern. The items will be scored on a seven-point Likert-type scale ranging from 0 to 6. The scores on the four subscales account for the EDE-Q total score, which will be the primary outcome measure in this study. Validation studies of the EDE-Q have shown a high level of agreement between the EDE-Q and EDE in assessing the core attitudinal features of eating disorder psychopathology in the general population [54,57], among female substance abusers [58], and in clinical samples of both BN and BED patients [59,60]. Luce & Crowther [61] and Reas, Grilo & Masheb [62] also demonstrated acceptable internal consistency and test-retest reliability.

### Secondary outcome measures

#### Body dissatisfaction

The Body Attitude Test (BAT) developed by Probst and colleagues [63-66] is a 20-item questionnaire measuring the subjective perception and attitude of the participant against her own body. The items will be scored on a six-point Likert-type scale ranging from 0 to 5. A higher score represents a more disordered body experience. The cut-off score between patients with body dissatisfaction and patients without body dissatisfaction is 36 [65].

#### Physical health

The physical complaints related to eating disorders will be assessed using the Maudsley Addiction Profile-Health Symptom Scale (MAP-HSS) [67]. This is a 10-item structured interview, which was adapted from the health scale of the Opiate Treatment Index [68]. Each item will be scored on a five-point Likert-type scale ranging from 0 (complaint never present in the previous 30 days) to 4 (complaint always present in the previous 30 days), resulting in a total scale-score ranging from 0 to 40. In previous research among opiate users, the scores on the MAP-HSS were approximately evenly distributed, the internal consistency of the scale was satisfactory (α = 0.79), and the test-retest reliability of the scale was high, with an intra-class correlation coefficient amounting to 0.86 [67]. Because the MAP-HSS only measures general physical complaints, 15 additional items (dizziness/fainting; insomnia; hoarseness; sore throat; palpitations; diarrhea; constipation; hair loss/brittle hair; downy hair on face, arms, chest or back; fluid accumulation in the legs; dry/scaly skin; rapidly cold; dental problems; damaged back of the hand; swollen glands) will be added to measure eating disorder-specific physical complaints. The total score of physical complaints will be determined by dividing the sum of the scores on the MAP-HSS and the additional items by the total number of items (n = 25).

#### Body weight

Body weight will be measured with one item: 'What is your current weight?’ Participants will be asked to fill in their body weight in kilograms.

#### Mental health

The Depression Anxiety and Stress Scale-21 (DASS-21) [69] is a 21-item self-report questionnaire assessing levels of depression, anxiety and stress. Participants will be asked to respond to each item to indicate the extent to which it applies to them in the previous week. Each item will be scored on a four-point scale ranging from 0 (did not apply to me at all) to 3 (applied to me very much, or most of the time). The DASS-21 has been shown to have high concurrent validity (r = 0.84) and reliability (i.e. Cronbach’s alpha of 0.94, 0.87 and 0.91 for the subscales depression, anxiety and stress, respectively) [70].

#### Self-esteem

The Rosenberg Self-Esteem Scale (RSES) [71] is a widely used self-report questionnaire consisting of 10-items to measure an individual’s overall self-esteem. Higher numbers represent higher levels of self-esteem. The RSES consists of 10 statements, of which five are negative and five positive. It has been demonstrated to be highly reliable with Cronbach’s alphas in the range of 0.77 to 0.88 [72].

#### Quality of life

The EuroQol-5D (EQ-5D) [73] is a standardized measure of health status, which can be used in the clinical and economic evaluation of health care. The EQ-5D consists of the ED-5D descriptive system and the EQ-5D visual analogue scale (EQ VAS). The EQ-5D descriptive system will measure quality of life on 5 dimensions: Mobility, Self-care, Usual activities, Pain/discomfort, and Anxiety/Depression. There are three levels of severity for each item: (1) no problems, (2) some problems, (3) severe problems. The scores on the 5 dimensions will be combined in a 5-digit number describing the participant’s global health state. A total of 243 possible health states are described. The EQ VAS will record the participant’s self-rated health on a visual analogue scale ranging from 0 ('Worst imaginable health state’) to 100 ('Best imaginable health state’).

#### Social contacts

Social contacts will be measured using three dimensions of the MATE (Measurements in the Addictions for Triage and Evaluation) part 7 'Activities & Participation, Care & Support’: (1) interpersonal interactions and relationships, (2) important areas of life, and (3) social life [74,75]. The MATE was constructed according to the ICD and International Classification of Functioning (ICF) in the World Health Organization (WHO) classification system. The dimension 'interpersonal interactions and relationships’ consists of 5 items, which will be scored on a five-point Likert-type scale, ranging from 1 (no / not applicable) to 5 (completely). The dimensions 'important areas of life’ and 'social life’ both consist of 2 items, using the same five-point severity scale.

#### Motivation for treatment

To measure motivation for treatment, the TCU Motivation for Treatment (MfT) [76] will be used. The MfT questionnaire was originally developed to measure treatment motivation in people with alcohol dependence, therefore, some textual adjustments will be made to make it suitable for patients with eating disorders.

#### Therapeutic alliance

The Helping Alliance Questionnaire (HAQ) is an 11-item self-report questionnaire to give a quick and global impression of the perception of the quality of the working alliance between the participant and the therapist [77,78]. The questionnaire contains two scales: (1) 'cooperation’, reflecting the participant’s perception of the measure of help received from the care provider; and (2) 'helpfulness’, referring to the participant’s confidence in his or her own capacity to improve the situation.

#### Participants’ acceptability of the web-based treatment program and satisfaction with their personal therapist

At post-treatment, the participants’ acceptability of the program and satisfaction with their own personal therapist will be measured. The participants will be asked to identify those aspects of the treatment which they consider to be most important, and to indicate how pleasant, personal, and safe they found the treatment program. The participants will also be asked to confirm whether the web-based treatment program was effective for them and whether they would recommend the intervention to others. The participants will also have to rate the treatment program and their own personal therapist on a scale from 0 (very low) to 10 (very high). Finally, they will have the opportunity to provide additional comments.

### Sample size and power calculation

The sample size is calculated with the statistical power analyses program “G-power 3.1” by using a significance level of 95% (p < .05), a power of 80%, the same number of participants per condition (M 1:1), 2 groups, 2 measurements, a correlation among repeated measures of 0.95, a mean difference (MD) score of 1.0, and a standard deviation (SD within group) of 1.2. The MD and SD are based on the results of the EDE-Q total score during our first study among 28 participants (results have not been published). Based on this calculation 25 participants per condition (experimental treatment group, waiting list control group) will be needed. However, some participants will end the treatment program prematurely. In the first study this amounted to 46% of the participants. We expect this will be less during the RCT, given the inclusion and exclusion criteria, the written informed consent, and the completers rate within the RCT of Ruwaard & Lange [37]. We expect that at least 60% of the RCT participants will complete all treatment sessions of the web-based treatment program. This means that 42 participants per condition are needed. As we want to measure the effectiveness of the web-based treatment program for the specific eating disorder subtypes BN, BED and EDNOS, we will need 42 participants per condition for all of the individual subtypes. This brings the total number to 252 participants.

### Randomization

The participants will be randomized to either the experimental treatment group or the waiting list control group via computer-generated randomly varying block sizes (2, 4 or 8), stratified by type of eating disorder (BN, BED, EDNOS). Block randomization will ensure that equal numbers of participants will be recruited into each subgroup (respectively, 42 participants in the BN experimental group, BN control group, BED experimental group, BED control group, EDNOS experimental group, and EDNOS control group). The computer-generated randomization will be prepared by a research assistant who is not involved in either testing or implementing the intervention. The lead investigator will be responsible for the allocation of the participants to one of the two groups. She will contact the research assistant every time a new participant can be allocated to one of the two groups, in order to ask the research assistant to which group the next participant should be assigned. The lead investigator will then assign the participant to that group via the web-based application. The participant will receive an automatic email which will indicate the group to which she has been assigned.

### Statistical analysis

All study data will be collected online on a secured database server, which will be accessible only via firewall and webserver through an SSL-secured (Secure Sockets Layer) connection. All data will be stored daily as an encrypted backup. Encryption keys for this backup will be changed every six months. Frequency tables will be provided for all baseline, end of treatment, and follow-up variables. Descriptive statistics will include mean, median, and numbers of participants. If applicable, 95% confidence intervals will be given. Chi-square and *t*-tests will be used for demographic data and pre-treatment characteristics, to assess whether randomization resulted in two comparable groups at baseline and whether any differential loss to follow-up has occurred. All participants of the study will complete the intake questionnaire during the signing-up stage. It is technically impossible to skip any of the questions in the questionnaires. Therefore, pre-treatment data will be available from all of these participants.

We will perform intention-to-treat analyses using repeated measures analyses (Mixed Models) to measure the effectiveness of the web-based treatment program in terms of primary and secondary outcomes. Mixed Model Repeated Measures will allow for the inclusion of all the participants over time, regardless of missing data. Between-group effect sizes will be calculated based on the pooled standard deviation. Effect sizes of *d* = .80 will be considered large [79]. Additionally, post-hoc analyses will be used to compare the effects of the web-based treatment program between the different groups of participants (BN, BED and EDNOS).

Linear regression analyses will be used to determine the predictors of improvement in eating disorder psychopathology and logistic regression analyses will be used to determine the predictors of premature termination of the web-based treatment program.

### Ethical approval

The study protocol was approved by the Ethics Committee of Medical Spectrum Twente in March 2011 (reference number NL31717.044.010, P10-31).

## Discussion

The present study protocol presents the design of a randomized controlled trial (RCT) to evaluate the effectiveness of a web-based treatment program for female patients with BN, BED and EDNOS. The main purpose of the treatment program will be to improve eating disorder psychopathology. The treatment will also aim to improve body image, physical health, body weight, mental health, self-esteem, quality of life, and social contacts. The participants will be followed for up to 12 months after completing the web-based treatment program in order to assess the short and long-term effectiveness of the program. Evaluation results will provide the support necessary to design treatments that are accessible, low-threshold and effective for female patients with BN, BED and EDNOS. The results of the trial will be published according to the CONSORT statement [80].

## Abbreviations

AN: Anorexia nervosa; BAT: Body attitude test; BED: Binge eating disorder; BN: Bulimia nervosa; DASS-21: Depression anxiety and stress scale-21; EDE-Q: Eating disorder examination questionnaire; EDNOS: Eating disorder not otherwise specified; EQ-5D: EuroQol-5D; HAQ: Helping alliance questionnaire; MAP-HSS: Maudsley addiction profile–health symptom scale; MfT: TCU Motivation for treatment questionnaire; SSL: Secure sockets layer; RCT: Randomized controlled trial; RSES: Rosenberg self-esteem scale.

## Competing interests

The authors declare that they have no competing interests.

## Authors’ contributions

All of the authors contributed to the design and development of the study. EtH was primarily responsible for drafting the manuscript under the supervision of MP, HdH, and CdJ. EtH is responsible for collecting the data, data analysis, and reporting the results of the study. All of the authors read and approved the final manuscript.

## Pre-publication history

The pre-publication history for this paper can be accessed here:

http://www.biomedcentral.com/1471-244X/13/310/prepub
